# Andrographolide Protects against LPS-Induced Acute Lung Injury by Inactivation of NF-κB

**DOI:** 10.1371/journal.pone.0056407

**Published:** 2013-02-21

**Authors:** Tao Zhu, Dao-xin Wang, Wei Zhang, Xiu-qing Liao, Xian Guan, Hong Bo, Jia-yang Sun, Ni-wen Huang, Jing He, Yun-kun Zhang, Jing Tong, Chang-yi Li

**Affiliations:** 1 Respiratory Medicine, Second Affiliated Hospital of Chongqing Medical University, Chongqing, China; 2 Respiratory Medicine, First Affiliated Hospital of Chengdu Medical College, Chengdu, China; 3 Respiratory Medicine, Chongqing Fuling Central Hospital, Chongqing, China; 4 Nephrology Medicine, West China Hospital, Sichuan University, Chengdu, China; 5 Respiratory Medicine, Affiliated Hospital of Guiyang Medical College, Guiyang, China; Harvard Medical School, United States of America

## Abstract

**Background:**

Nuclear factor-κB (NF-κB) is a central transcriptional factor and a pleiotropic regulator of many genes involved in acute lung injury. Andrographolide is found in the plant of *Andrographis paniculata* and widely used in Traditional Chinese Medicine, exhibiting potently anti-inflammatory property by inhibiting NF-κB activity. The purpose of our investigation was designed to reveal the effect of andrographolide on various aspects of LPS induced inflammation in vivo and in vitro.

**Methods and Results:**

In vivo, BALB/C mice were subjected to LPS injection with or without andrographolide treatments to induce ALI model. In vitro, MLE-12 cells were stimulated with LPS in the presence and absence of andrographolide. In vivo, pulmonary inflammation, pulmonary edema, ultrastructure changes of type II alveolar epithelial cells, MPO activity, total cells, neutrophils, macrophages, TNF-α, IL-6 and IL-1β in BALF, along with the expression of VCAM-1 and VEGF were dose-dependently attenuated by andrographolide. Meanwhile, in vitro, the expression of VCAM-1 and VEGF was also reduced by andrographolide. Moreover, our data showed that andrographolide significantly inhibited the ratios of phospho-IKKβ/total IKKβ, phospho-IκBα/total IκBα and phospho-NF-κB p65/total NF-κB p65, and NF-κB p65 DNA binding activities, both in vivo and in vitro.

**Conclusions:**

These results indicate that andrographolide dose-dependently suppressed the severity of LPS-induced ALI, more likely by virtue of andrographolide-mediated NF-κB inhibition at the level of IKKβ activation. These results suggest andrographolide may be considered as an effective and safe drug for the potential treatment of ALI.

## Introduction

*Andrographis panicula* a popular herb has been widely used as folk medicine in China for the treatment of viral infection, diarrhoea, dysentery and fever for hundreds of years. Moreover, andrographolide, extracted and purified from *Andrographis panicula*, is currently prescribed for treatment of inflammation related diseases, such as laryngitis, upper respiratory tract infection and rheumatoid arthritis in China [1]–[7]. Recently, several studies have showed that the anti-inflammatory properties of andrographolide were resulted from inactivation of NF-κB at the level of the phosphorylation of IKKβ [1], [8], [9]. Otherwise, a clinical study found that rheumatoid arthritis symptoms were relieved after andrographolide treatment [10].

Acute lung injury (ALI) and its severe form, acute respiratory distress syndrome (ARDS), were the diseases induced by many extreme conditions including severe sepsis, severe bacterial pneumonia, trauma and burn. The pathophysiological mechanism of ARDS is believed to be associated with the uncontrolled inflammatory response in lungs [11]–[13]. Meanwhile, many studies found that the NF-κB pathway played a pivotal role on ALI [14]–[17]. The NF-κB family of transcriptional factors (TF) plays a critical role on regulation the expression of a wide variety of genes, particularly in the inflammatory process. The NF-κB family has five cellular members. They are p105/p50 (NF-κB1), p100/p52 (NF-κB2), p65 (RelA), RelB and c-Rel [1], [18], [19]. The activation of NF-κB pathway was tightly dependent on the IKK (IκB kinase) complex, which consists of the regulatory subunit, IKKγ also called NF-κB essential modifier (NEMO), and the catalytic subunits, IKKα and IKKβ. And its downstream substrate is IκBα. In unstimulated cells, NF-κB p50/NF-κB p65 dimer is bound to IκBα. After the stimulation, IKKα and IKKβ are phosphorylated, then, promoting the phosphorylation of IκBα. Rapidly, phosphorylated IκBα is degraded via the ubiquitin-proteasome pathway. The degradation of IκBα leads to NF-κB p50/NF-κB p65 dimer phosphorylation and translocation, resulting in the transcription of genes, finally [1], [18], [19].

Vascular adhesion molecule (VCAM)-1, a member of the immunoglobulin gene superfamily, is up-regulated in inflammatory condition, promoting the inflammatory cells binding to the endothelial cell [20]. Vascular endothelial growth factor (VEGF), another important inflammatory mediator, is one of main contributor of lung angiogenesis and regulator of vascular permeability which is the critical pathological process in ALI associated pulmonary edema [21], [22]. Recently, the study showed that NF-κB played a critical role in VCAM-1 gene regulation by binding its promoter [23]. Moreover, further investigation found that PI3K-Akt dependent NF-κB signaling pathway was essential to the expression of VCAM-1 in MLE-12 [20]. Meanwhile, it has been found that the activation of NF-κB leads to the expression of VEGF [24]. Then, another report also indicated that deletion of NF-κB binding site, but not HRE or binding sites for AP-1 and AP-2, completely abolished hypoxia-induced mitogenic factor (HIMF)-induced VEGF promoter activity in MLE-12 cells [21]. According to these data, VEGF and VCAM-1 were used in our study to observe the effect of andrographolide on the inhibition of NF-κB in in vivo and in vitro.

Therefore, our study was designed to investigate the value of andrographolide on various aspects of LPS induced inflammation in vivo and in vitro.

## Materials and Methods

### Animals

All animal use procedures were approved the Committee on the Ethics of Animal Experiments of Chongqing Medical University. This investigation was carried out in strict accordance with the recommendations in the Guide for the Care and Use of Laboratory Animals of the National Institutes of Health. All surgery was performed under sodium pentobarbital anesthesia, and all efforts were made to minimize suffering. Six weeks old specific pathogen-free male BALB/c mice (16–20 g) were maintained under specific pathogen-free conditions in the facilities of the animal center in our university. The mice were kept in a temperature controlled room with 12 h dark/light cycles, and allowed food and water ad libitum. Animals underwent an acclimatization period of at least 7 days before use in our study.

### Murine Model of LPS-induced ALI

Forty male BALB/c mice were randomly divided into 4 groups (n = 10), control group, LPS group, low dosage andrographolide (Sigma, St. Louis, MO, USA) treatment group (1 mg/kg, LPS+Andro-L group) and high dosage andrographolide treatment group (10 mg/kg, LPS+Andro-H group). According to the report, ALI was induced by LPS (E.coli LPS serotype 0111: B4, Sigma, St. Louis, MO, USA) via intratracheal injection [23]. In brief, mice were anesthetized with pentobarbital sodium (30 mg/kg), followed by 10 µg LPS in 50 µL sterile saline intratracheal injection with a 3-gauge needle. The mice in control group were administrated the sterile saline instead. Then, the mice were placed in a vertical position and rotated for 1 minute to distribute the instillation in the lungs. Ten minutes after LPS injection, andrographolide was given by intraperitoneal injection at 1 mg/kg in 100 µL PBS (LPS+Andro-L group) or 10 mg/kg in 100 µL PBS (LPS+Andro-H group). The next 2 days, the mice in treatments group received andrographolide injection per 12 hours.

### Bronchoalveolar Lavage Fluid (BALF) and Cells Counting

Seventy two hours later, mice were exsanguinated after pentobarbitone (50 mg/kg i.p.) anesthesia. According to the previous report, BALF was collected by cannulating the upper part of the trachea, by lavage three times with 1.0 ml PBS (pH 7.2). The fluid recovery rate was more than 90%. Lavaged sample from each mouse was kept on ice. BALF was centrifuged at 700 ×g for 5 min at 4°C [23]. The sediment cells were resuspended in 50 µL PBS and stained with Diff-Quik (International Reagents Corp., Japan) for cytospin preparations. Then, total cells, neutrophils, macrophages and lymphocytes were counted double-blindly with hemocytometer.

### TNF-α, IL-6 and IL-1β in BALF

The BALF supernatant was collected after centrifugation (for 4 minutes at 4000 rpm) and stored at –70°C before cytokine assay. TNF-α, IL-6 and IL-1β in BALF were measured by ELISA (R&D Systems, USA). The limit of detection of this method was better than 7.8 pg/ml.

### Myeloperoxidase (MPO) Activity Assay

After BALF collection, the left upper lobe was removed, washed and kept in –80°C. Then, after weighing, the lungs were homogenized, centrifuged (40,000 rpm, 30 min, 4°C) and resuspended in 50 mM KPO_4_ buffer (PH 6.0) with 0.5% hexadecyltrimethylammonium bromide (HTAB). Then, samples were sonicated and incubated at 60°C for 2 h. Later, samples were assayed for activity in a H_2_O_2_/*o*-dianisidine buffer at 460 nm with a spectrophotometer (Shanghai Precision & Scientific Instrument Co. Ltd, China). Results are expressed as units of MPO activity per gram of lung tissue.

### Lung Wet/dry Weight Ratio

The severity of pulmonary edema was assessed by the wet to dry ratio (W/D ratio). The right lower lungs weighed and then dehydrated at 60°C for 72 h in an oven.

### H&E Staining

The left lower lung from each mouse was fixed in 10% formalin, embedded in paraffin, cut into 5 µm sections, stained with H&E. Lung injury score was measured by a blinded pathologist with a 0 to 4 point scale according to combined assessments of inflammatory cell infiltration in the airspace or vessel wall, alveolar congestion, hemorrhage, alveolar wall thickness and hyaline membrane formation. A score of 0 represented no damage; l represented mild damage; 2 represented moderate damage; 3 represented severe damage and 4 represented very severe histological changes [24].

### Transmission Electron Microscope

The fresh left upper lung tissues (2×2×2 mm) were taken for electron microscopy. The specimen was fixed in 2.5% glutaraldehyde and phosphate buffer. The specimen was then rinsed in phosphate buffer, postfixed with 1% osmic tetroxide in phosphate buffer. After graded dehydration in ethyl alcohol and propylene oxide, specimen was embedded in Spurr resin. Then, the embedded tissues were thin-sectioned, mounted on copper grids, and stained with uranyl acetate and lead citrate. The images were taken by electron microscope (H-600IV, HITACHI, Tokyo, Japan).

### Cell Culture and Treatments

MLE-12 cells (American Type Culture Collection [ATCC], CRL-2110), a cell line derived from murine alveolar epithelial cells, were plated at a density of 5×10^3^ cells per well in a 96-well plate and grown to 80% confluency in Hites media 10% fetal bovine serum. Then, cells were pretreated with 50 µM andrographolide or vehicle (0.01% DMSO) 4 h before LPS (0.5 µg/ml) stimulation. Total and nuclear proteins and mRNA were extracted from cells at specified time intervals. MTT assay (Promega, Madison, WI) was used to assess cell viability.

### RT-PCR (Real Time Reverse-transcribed Polymerase Chain Reaction)

Total RNA was isolated from the lung tissues and MLE-12 cells by Trizol reagent (Invitrogen, USA). PCR was performed with a DNA thermal cycler in a 50 µl reaction volume, containing 5 µl 10×Taq Buffer, 4 µl 2.5 mM dNTP, 4 µl 25 mM MgCl2, forward and backward primers 2 µl each, Taq polymerase 0.5 µl and cDNA template 2 µl, for 35 cycles via GeneAmp PCR system 9700 (Applied Biosystems, Foster City, CA). The primer sequences were as follows: VEGF (forward) 5′-TGGATGTCTACCAGCGAAGC-3′ and (reverse) 5′-ACAAGGCTCACAGTGATTTT-3′; VCAM-1 (forward) 5′-CCTCACTTGCAGCACTACGGGCT-3′ and (reverse) 5′-TTTTCC AATATCCTCAATGACGGG-3′ β-actin, (forward) 5′-CGAGCGGGCTACAGCTTC-3′ and (reverse) 5′-GTCACGCACGATTCCCTCT-3′. The mouse β-actin housekeeping gene was used as an internal control.

### Western Blotting

The protein expression was measured by western blotting. The lung tissues were kept in −80°C. By the aid of a tissue grinder, the lung tissues were homogenized in PBS containing the protease inhibitor cocktail. The homogenates were centrifuged for 15 min at 14, 000 rpm in 4°C. Meanwhile, cells were washed twice with cold PBS. Then, cells were lysed with SDS sample buffer containing 50 mM Tris (pH 7.4), 2% SDS (wt/vol), 5% 2-mercaptoethanol and 10% glycerol. Cell homogenates were centrifuged at 10,000 rpm at 4°C for 60 min. Supernatants of lung tissues and cells were collected, and protein concentration of each sample was measured with a bicinchoninic acid assay kit using BSA as standard (Pierce, Rockford, IL). An equal amount of protein from each sample (150 µg) was resolved in 10% Tris-glycine SDS polyacrylamide gel. Protein band was blotted to nitrocellulose membrane. After incubation for 1 h in blocking solution (5% dry milk in Tris-buffered saline with Tween 20) at room temperature (RT), the membrane was incubated for 24 h with anti-VEGF (1∶500 Santa Cruz Biotechnology, Inc.), anti-VCAM-1 (1∶500 Santa Cruz Biotechnology, Inc.), anti-β-actin (1∶1000 Santa Cruz Biotechnology, Inc.), anti-IKKβ (1∶800 Santa Cruz Biotechnology, Inc.), anti-phospho-IKKβ (1∶800 Santa Cruz Biotechnology, Inc.), anti-IκBα (1∶800 Santa Cruz Biotechnology, Inc.), anti-phospho-IκBα (1∶800 Santa Cruz Biotechnology, Inc.), anti-NF-κB p65 (1∶800 Santa Cruz Biotechnology, Inc.) or anti-phospho-NF-κB p65 (1∶800 Santa Cruz Biotechnology, Inc.), at 4°C, respectively. The secondary antibody (horseradish peroxidase-conjugated donkey anti-rabbit immunoglobulin) was added at 1∶10,000 dilution and incubated at room temperature for 1 h. Peroxidase labeling was detected with the enhanced chemiluminescence Western blotting detection system (Amersham Pharmacia Biotech) and analyzed by a densitometry system. The relative protein level was normalized to β-actin (Santa Cruz Biotechnology, Inc.).

### NF-κB p65 DNA Binging Activity Assay

For detection NF-κB p65 DNA-binding activity, TransAM™ NFκB p65 Chemi Transcription Factor Assay Kit was used (Active Motif, Carlsbad, CA).

### Statistical Analysis

Statistical analyses were performed with Sigmaplot software (SPSS). Each point corresponds to mean ± SD Statistical differences were determined by one-way or two-way analysis of variance (ANOVA) and *P*<0.05 was considered to be statistically significant.

## Results

### Andrographolide Attenuates Pulmonary Inflammation and Pulmonary Edema in vivo

**Figure 1:**
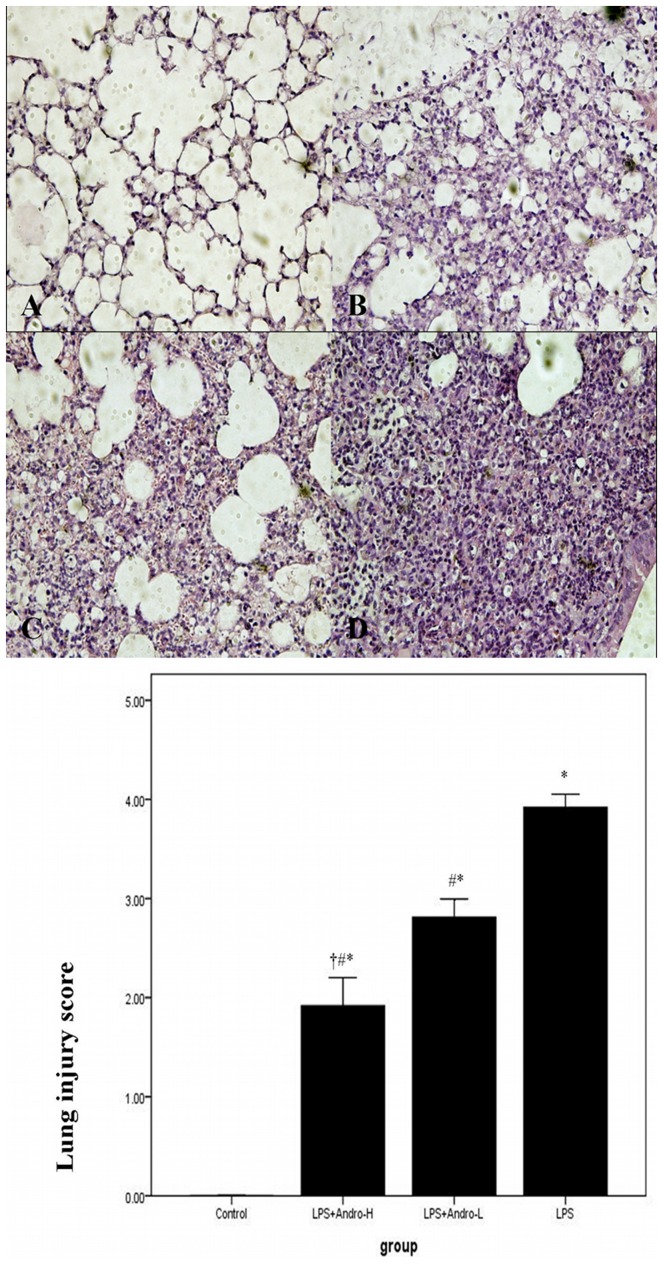
Andrographolide attenuates pulmonary inflammation in vivo. Seventy two hours after LPS injection with or without andrographolide treatments, mice were exsanguinated and their left lower lungs were fixed. Then, tissue sections were stained with hematoxylin and eosin (H&E). (A) The figure demonstrates a representative view (×400) from each group. [A: Control B: LPS+Andro-H C: LPS+Andro-L D: LPS]. (B) Degree of lung injury was measured via the lung injury scoring system. *P<0.01 compared with Control. ^#^ *P*<0.01 compared with LPS. ^†^ *P*<0.01 compared with LPS+Andro-L.

**Figure 2:**
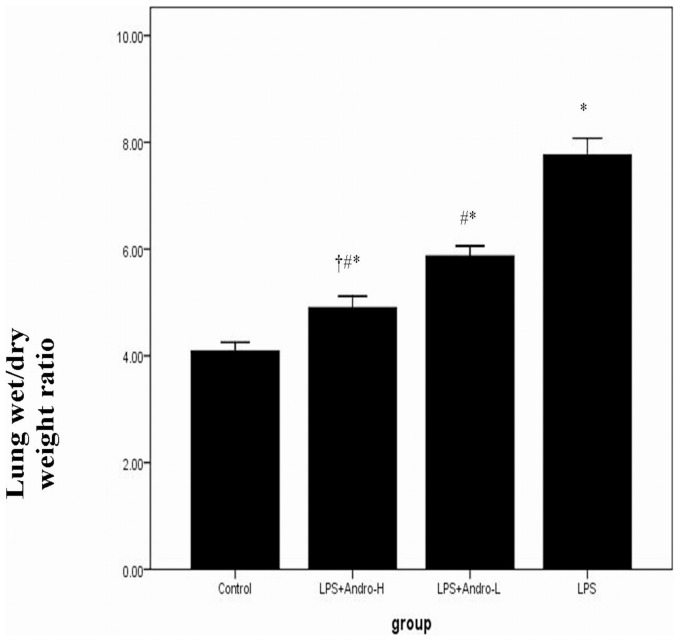
Andrographolide improves pulmonary edema in vivo. Seventy two hours after LPS injection with or without andrographolide treatments, mice were exsanguinated and their right lower lungs were obtained. The right lower lungs weighed and then dehydrated at 60°C for 72 h in an oven. Each bar represents the mean ± SD of 10 mice. *P<0.01 compared with Control. ^#^ *P*<0.01 compared with LPS. ^†^ *P*<0.01 compared with LPS+Andro-L.

[LOOSEST ]To assess the pathological changes, HE staining and lung injury score system were used in our study. Histological evaluation of lung sections 3 day after the LPS injection revealed evidence of notable inflammatory cells infiltration, interstitial edema, interalveolar septal thickening and intraalveolar and interstitial patchy hemorrhage. However, after andrographolide treatments, the pathological changes in the lung tissues were relieved. (Fig. 1) And the mice in control group showed no histological changes. As for pulmonary edema evaluation, lung weight and dry ratio (W/D) was employed. The mice received LPS injection showed higher W/D than in controls. Nevertheless, W/D significantly decreased after andrographolide administrations. (Fig. 2) Additionally, the effect of andrographolide in pulmonary inflammation and pulmonary edema was in a dose-dependent manner.

### Andrographoilde Attenuates LPS-induced Type II Alveolar Epithelial Cell Ultrastructure Pathological Changes

**Figure 3:**
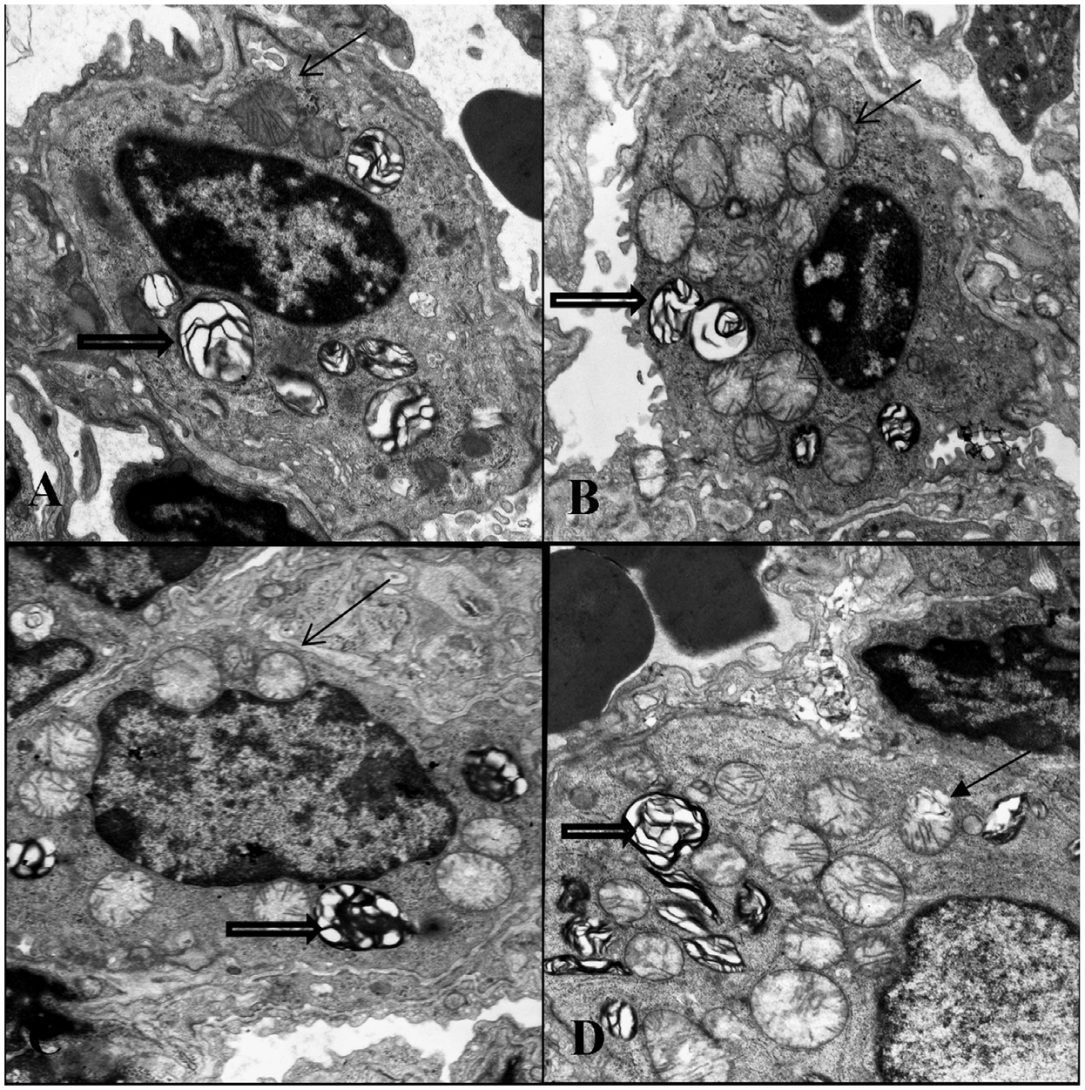
Andrographoilde attenuates LPS-induced type II alveolar epithelial cell ultrastructure pathological changes. After mice sacrificed, the left upper lung tissues were taken and observed under transmission electron microscope. The figure demonstrates a representative view (×8000) from each group. [A: Control B: LPS+Andro-H C: LPS+Andro-L D: LPS]. Mitochondrion (arrow); lamellar body (open arrow).

After 72 h LPS stimulation, typical changes were found in type II alveolar epithelial cells. These features included cell swollen, reduced and indistinct cell surface microvilli, mitochondria reduced with dilated mitochondrial cristae, along with decreased and vacuolated lamellar bodies. (Fig. 3D) However, the ultrastructure changes were attenuated by andrographolide. (Fig. 3B and 3C).

### Andrographolide Reduces Cellular Counts in BALF

**Figure 4:**
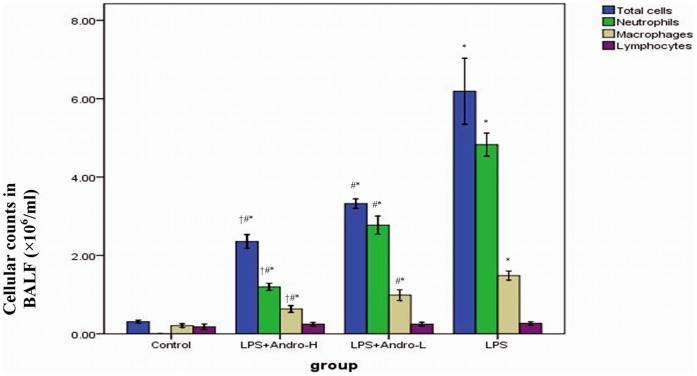
Andrographolide reduces cellular counts in BALF. Three days after LPS injection with or without andrographolide treatments, mice were sacrificed and their lungs were lavaged. Cells in the BALF were collected and cytospin preparations were made. Total cells, neutrophils, macrophages and lymphocytes in BALF were analyzed. Each bar represents the mean ± SD of 10 mice. *P<0.01 compared with Control. ^#^ *P*<0.01 compared with LPS. ^†^ *P*<0.01 compared with LPS+Andro-L.

To further identify the anti-inflammatory property of andrographolide, cellular counts in BALF were measured in our study. Three days after LPS injection, total cells, neutrophils and macrophages were remarkably increased in BALF. However, andrographolide treatments dose-dependently reduced total cells, neutrophils and macrophages in BALF. (Fig. 4).

### Andrographolide Down-regulates TNF-α, IL-6 and IL-1β in BALF

**Figure 5:**
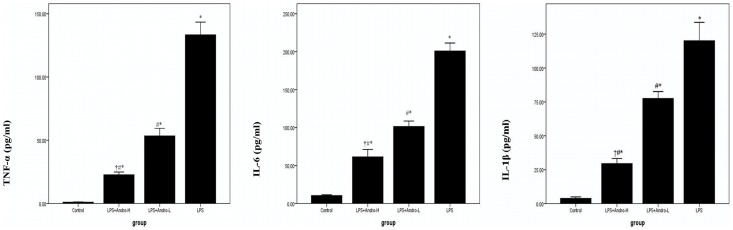
Andrographolide down-regulates TNF-α, IL-6 and IL-1β in BALF. Three days after LPS injection with or without andrographolide treatments, mice were sacrificed, their lungs were lavaged and the BALF were collected. TNF-α, IL-6 and IL-1β were detected by ELISA. Each bar represents the mean ± SD of 10 mice. *P<0.01 compared with Control. ^#^ *P*<0.01 compared with LPS. ^†^ *P*<0.01 compared with LPS+Andro-L.

TNF-α, IL-6 and IL-1β in BALF were measured by ELISA. TNF-α, IL-6 and IL-1β in BALF in the mice with LPS injection were markedly higher than the mice in control group. However, TNF-α, IL-6 and IL-1β in BALF in the mice with andrographolide injections were decreased. (Fig. 5).

### Andrographolide Reduces MPO Activity in vivo

**Figure 6:**
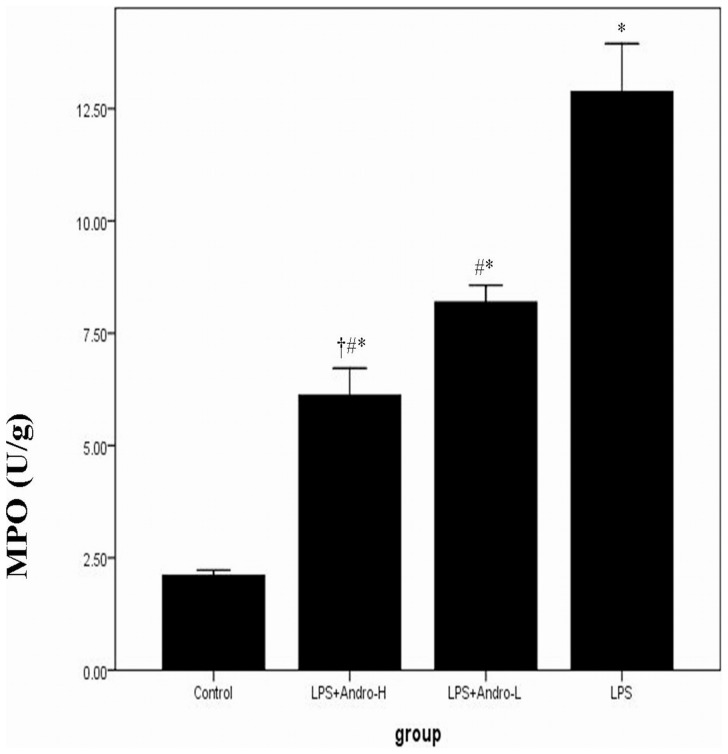
Andrographolide reduces MPO activity in vivo. Three days after LPS injection with or without andrographolide treatments, mice were sacrificed, their lungs were removed. MPO activity was detected by way described in Materials and Methods. Each bar represents the mean ± SD of 10 mice. *P<0.01 compared with Control. ^#^ *P*<0.01 compared with LPS. ^†^ *P*<0.01 compared with LPS+Andro-L.

The accumulation of activated of neutrophils was assessed by measuring MPO activity in the lung tissues. MPO activity in model group was markedly higher than control group. However, MPO activity was dose-dependently reduced by andrographolide. (Fig. 6).

### Andrographolide Inhibits VCAM-1 and VEGF Expression in vivo

**Figure 7:**
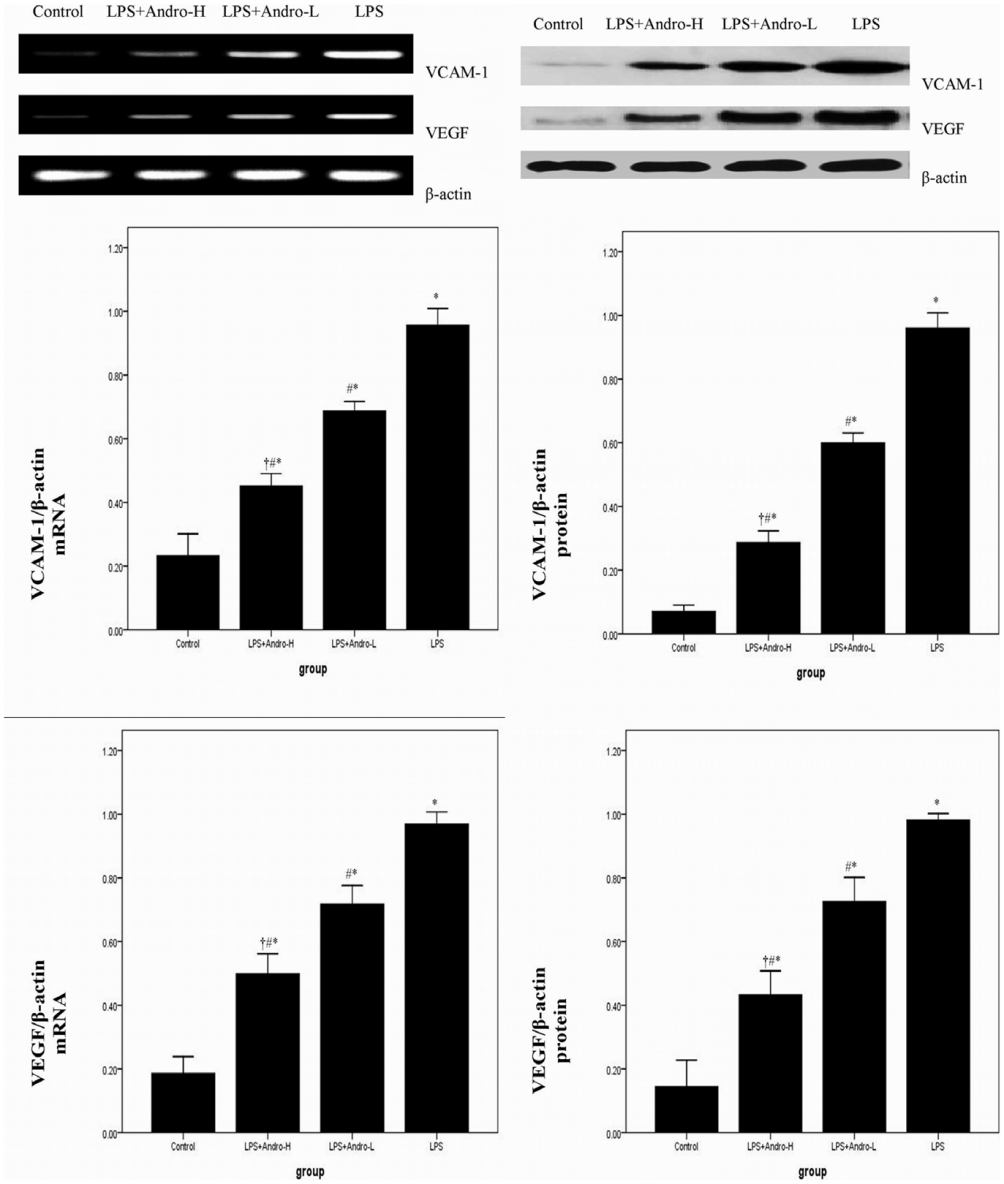
Andrographolide inhibits VCAM-1 and VEGF expression in lung. Seventy two hours after LPS injection with or without andrographolide treatments, mice were exsanguinated and their lungs were removed. (A) RT-PCR was performed to detect VCAM-1 and VEGF mRNA expression in the lung tissues. VCAM-1 and VEGF mRNA level in each sample was expressed as a ratio of VCAM-1 or VEGF gray value to β-actin. (B) Western blotting was performed to detect VCAM-1 and VEGF protein expression in the lung tissues. VCAM-1 and VEGF protein level in each sample was calculated as ratios of intensities of VCAM-1 or VEGF to the corresponding β-actin bands. Each bar represents the mean ± SD of 10 mice. *P<0.05 compared with Control. ^#^ *P*<0.05 compared with LPS. ^†^ *P*<0.05 compared with LPS+Andro-L.

Compared with control group, enhanced mRNA and protein expression of VCAM-1 and VEGF were noticed after LPS injection. (Fig. 7) Meanwhile, fig 7 A and fig. 7 B also showed that LPS-stimulated enhanced VCAM-1 and VEGF expression was dose-dependently inhibited by andrographolide.

### Andrographolide Inhibits NF-κB p65 Activation and DNA Binding Activity in vivo

**Figure 8:**
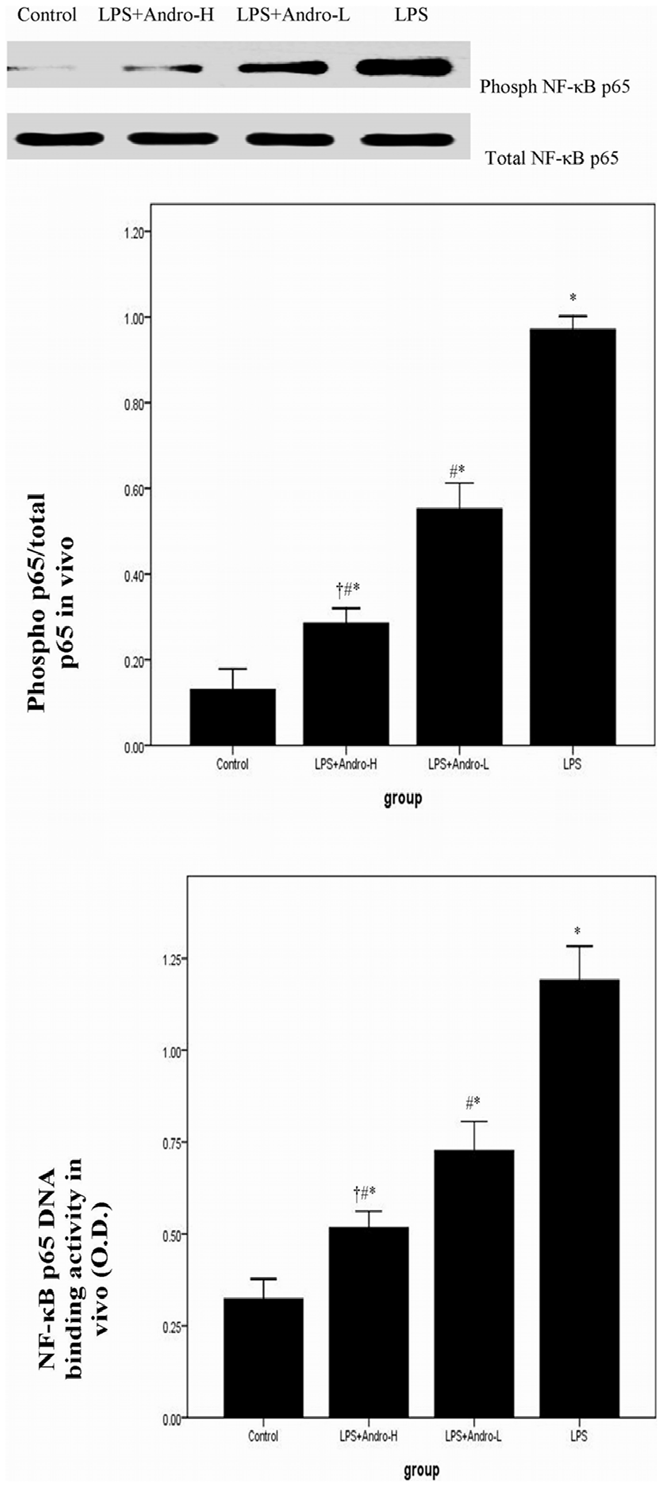
Andrographolide inhibits NF-κB p65 activation and DNA binding activity in vivo. Three days after LPS injection with or without andrographolide treatments, mice were sacrificed, their lungs were removed. (A) The lung tissues were subjected to western blotting analysis using anti-phospho-NF-κB p65 and anti-NF-κB p65. The levels of phospho-NF-κB p65 in each sample were measured as ratios of intensities of phospho-NF-κB p65 to total NF-κB p65 bands. (B) DNA binding activity of NF-κB p65 was examined by a TransAM p65 transcription factor ELISA kit. Each bar represents the mean ± SD of 10 mice. *P<0.01 compared with Control. ^#^ *P*<0.01 compared with LPS. ^†^ *P*<0.01 compared with LPS+Andro-L.

NF-κB is a critical transcription factor for inflammatory genes expression. Activation of NF-κB pathway leads to phophorylation of p50 and p65. Our investigation found a noticeable increased p65 subunit phophorylation 72 h after LPS injection, which was ameliorated by andrographolide, indicating that andrographolide inhibited the activation of NF-κB. (Fig. 8A) Meanwhile, NF-κB p65 DNA-binding activity represented NF-κB p65 subunit translocation. Our data showed that NF-κB p65 DNA-binding activity was significantly increased 72 h after LPS injection. However, the over DNA-binding activity of NF-κB p65 was dose-dependently abolished by andrographolide. (Fig. 8B).

### Andrographolide Improves Cell Viability

**Figure 9:**
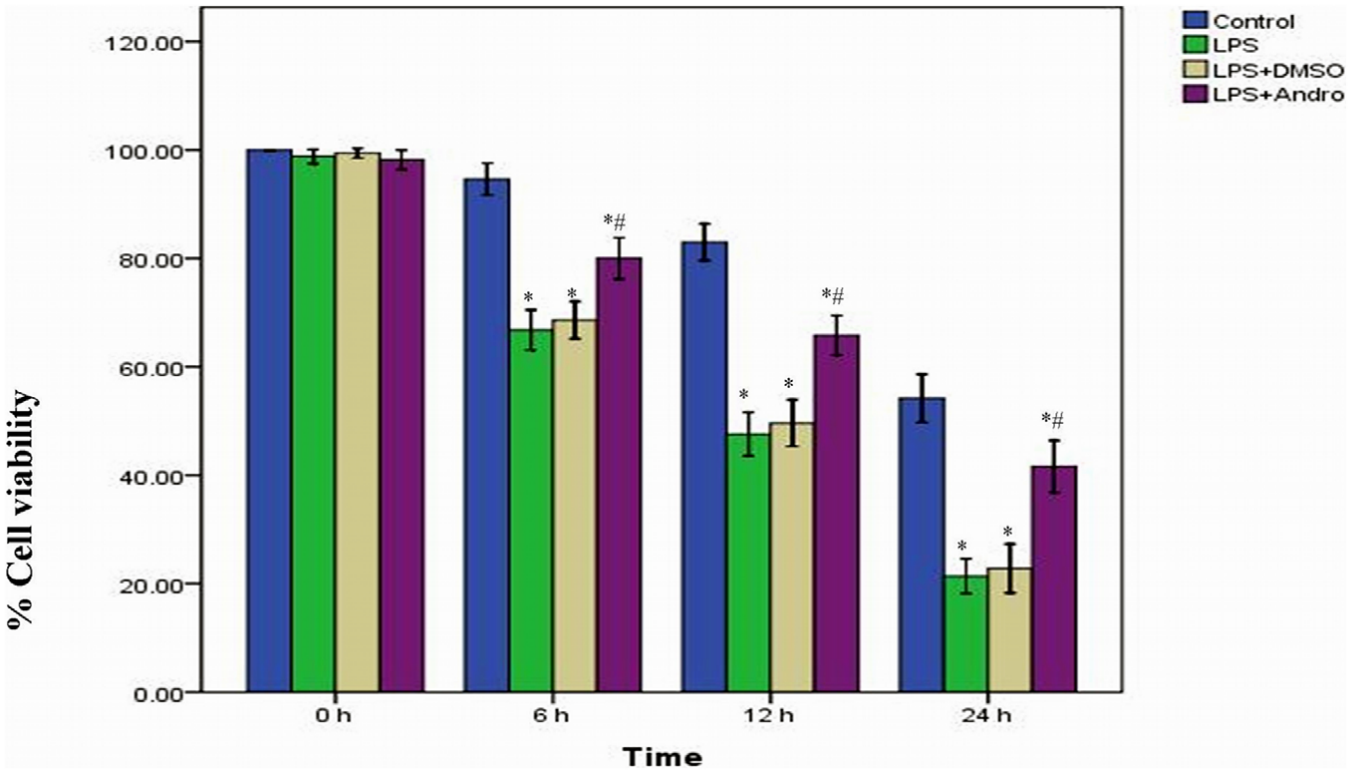
Andrographolide improves cell viability. MLE-12 cells were stimulated with 0.5 µg/ml LPS in the presence and absence of 50 µM andrographolide. The quantitative data were presented as mean ± SD (n = 5). *P<0.01 compared with Control at the corresponding time points. ^#^ *P*<0.01 compared with LPS at the corresponding time points.

MLE-12 cells, derived from murine alveolar epithelial cells, were used in our study. The cell viabilities were significantly reduced after LPS treatment. Meanwhile, MTT assay also revealed that the cell viabilities were improved in the presence of andgrapholide. (Fig. 9).

### Andrographolide Reduces VCAM-1 and VEGF in vitro

**Figure 10:**
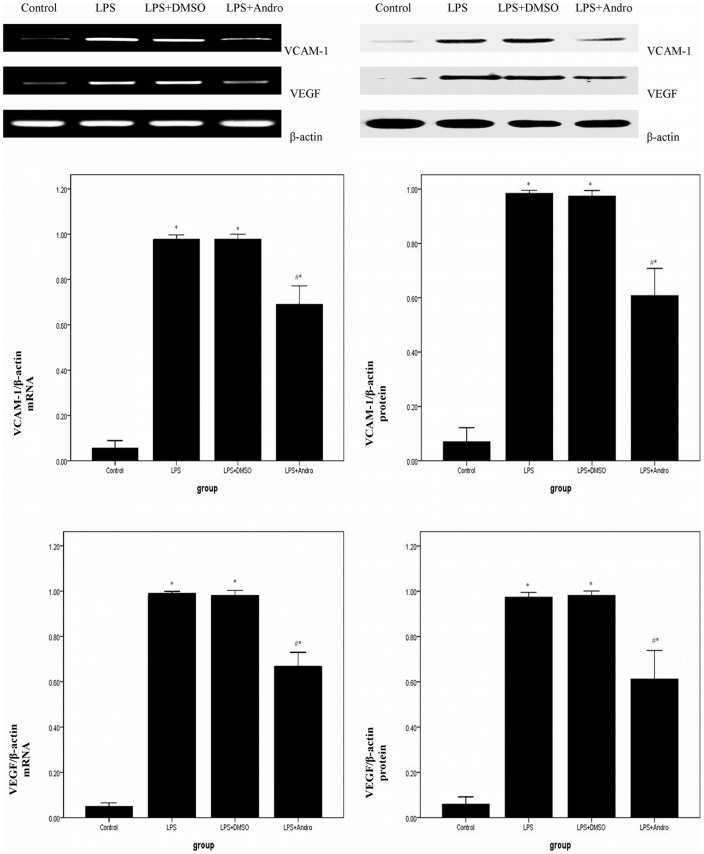
Andrographolide reduces LPS-induced inflammatory gene expression in vitro. MEL-12 cells were stimulated with 0.5 µg/ml LPS in the presence and absence of 50 µM andrographolide for 12 hours. (A) RT-PCR was performed to detect VCAM-1 and VEGF mRNA expression in vitro. VCAM-1 and VEGF mRNA level in each sample was expressed as a ratio of VCAM-1 or VEGF gray value to β-actin. (B) Western blotting was performed to detect VCAM-1 and VEGF protein expression in vitro. VCAM-1 and VEGF protein level in each sample was calculated as ratios of intensities of VCAM-1 or VEGF to the corresponding β-actin bands. Each bar represents the mean ± SD (n = 5). *P<0.01 compared with Control. ^#^ *P*<0.01 compared with LPS.

To further explore anti-inflammatory activity of andrographolide, MLE-12 cells were treated with LPS to induce inflammation reaction and NF-κB activation. LPS stimulation led to up-regulation of VCAM-1 and VEGF. Pretreatment with andrographolide (50 µM) exhibited strongly suppression of LPS-induced up-regulation of VCAM-1 and VEGF in vitro. (Fig. 10).

### Andrographolide Inhibits LPS-induced NF-κB Activation and DNA Binding Activity in vitro

**Figure 11:**
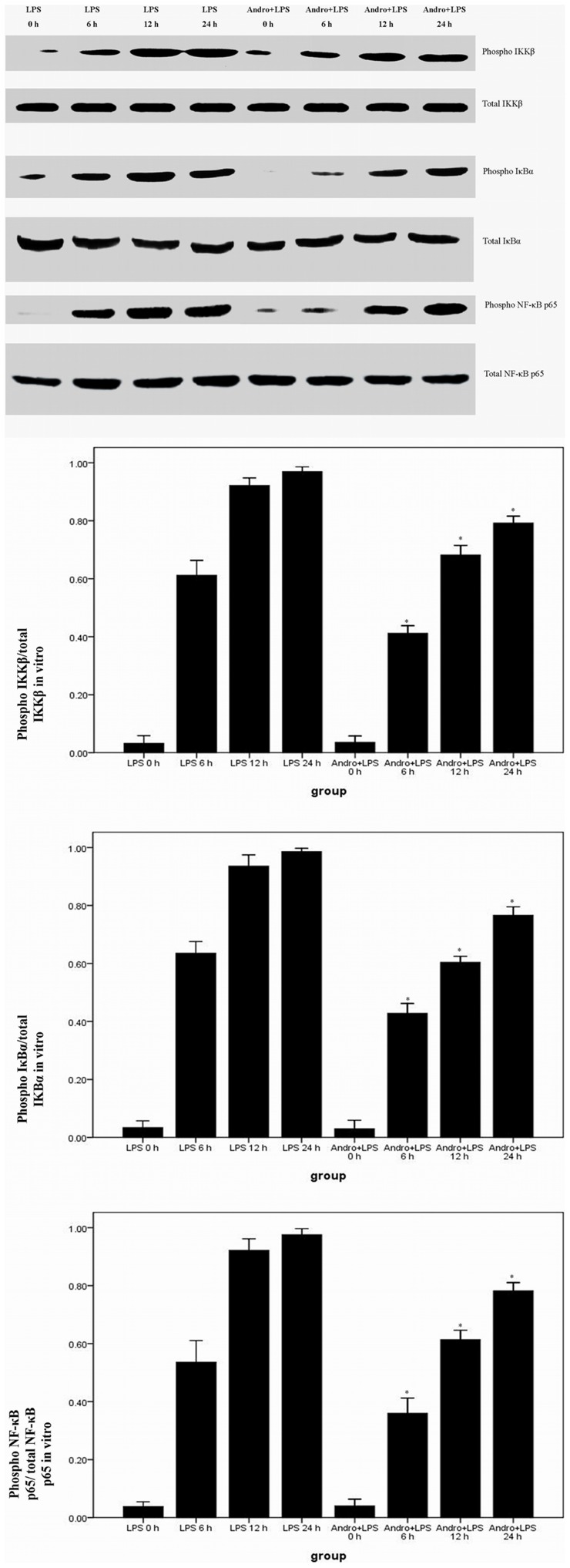
Andrographolide inhibits NF-κB p65 activation in vitro. MEL-12 cells were stimulated with LPS (0.5 µg/ml) in the presence and absence of 50 µM andrographolide for 0, 6, 12 and 24 hours before total proteins were extracted for subsequent western blotting. Immunoblots were probed with anti-IKKβ, anti-phospho-IKKβ, anti-IκBα, anti-phospho-IκBα, anti-NF-κB p65 and anti-phospho-NF-κB p65. The ratios of phospho-IKKβ/total IKKβ, phospho-IκBα/total IκBα and phospho-NF-κB p65/total NF-κB p65 were measured by densitometry system (Amersham Pharmacia Biotech). The quantitative data were presented as mean ± SD (n = 5). *P<0.05 compared with LPS at the corresponding time points.

**Figure 12:**
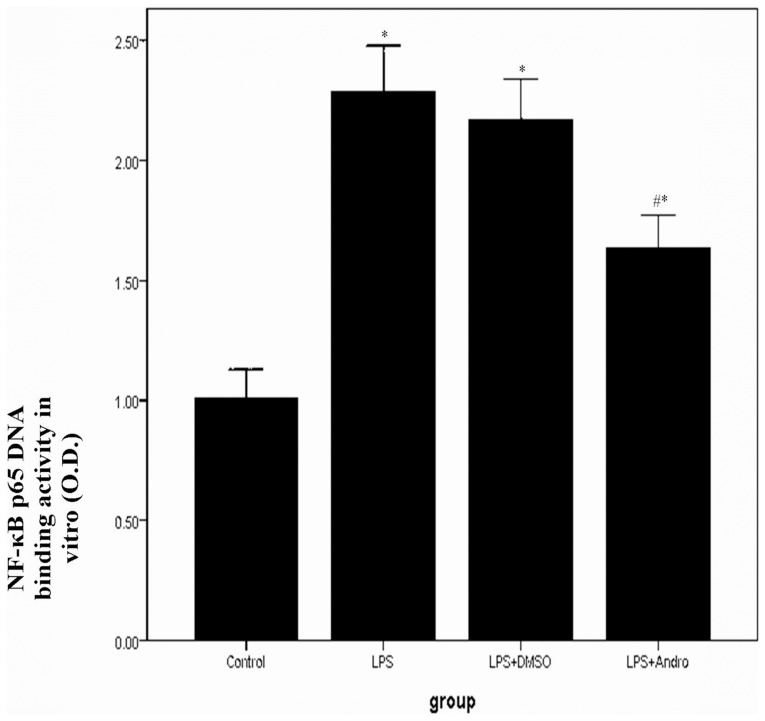
Andrographolide reduces NF-κB p65 DNA binding activity in vitro. MLE-12 cells were stimulated with 0.5 µg/ml LPS in the presence and absence of 50 µM andrographolide for 12 hours. Then, DNA binding activity of NF-κB p65 was determined by a TransAM p65 transcription factor ELISA kit. Each bar represents the mean ± SD (n = 5). *P<0.01 compared with Control. ^#^ *P*<0.01 compared with LPS.

LPS induced a rapid activation of IKKβ, IΚBα and NF-κB p65 in 6 h. And the maximum phosphorylation levels of IKKβ, IΚBα and NF-κB p65 were observed after 12 h of LPS stimulation. Meanwhile, after 24 h of LPS stimulation, the phophorylation levels of IKKβ, IΚBα and NF-κB p65 were still in maximum. In contrast, after andrographolide treatment, lower phophorylation levels of IKKβ, IΚBα and NF-κB p65 were observed at each time point. (Fig. 11) Furthermore, 12 hours after LPS stimulation markedly promoted NF-κB p65 DNA binding activity. However, a lower DNA binding activity was observed in MEL-12 cells with LPS stimulation in the presence of andrographolide. (Fig. 12).

## Discussion

In present study, we demonstrated that andrographolide dose-dependently attenuates LPS-induced ALI by inactivation of NF-κB at the level of the phosphorylation of IKKβ.

Andrographolide was one of major effective component in the plant of *Andrographis paniculata* and wildly used in China and other Asia countries. Recently, it was reported that andrographolide was a powerful NF-κB inhibitor in inflammatory diseases [1], [8], [9].

Acute respiratory distress syndrome (ARDS) is an acute and progressive respiratory disease without abnormal cardiac filling pressure and is characterized by progressively diffuse bilateral pulmonary edema and inflammation, reduced pulmonary compliance, and hypoxemia [11]–[13], [25]–[27]. Patients with ALI induced by different illnesses through a similar pathophysiological process irrespective of whether the damage is a direct effect on the alveolar epithelial cells by an external stimuli or an indirect process resulting from a more distant systemic inflammatory process mediated via cytokines. Endothelial injury and epithelial injury are the two major pathophysiological mechanisms of ALI in different etiologies. Pulmonary inflammation and pulmonary edema are two of most important pathological findings in ALI [11]–[13], [25]–[27].

LPS, the outer membrane of Gram-negative bacteria, was the one of mainly pro-inflammatory reaction factor in infection diseases, leading to over inflammatory reaction in vivo. Neutrophils and macrophages were the mainly inflammatory cells in ALI. They infiltrated into the lung tissues, releasing enzymes and phagocytizing the pathogen. Meanwhile, these inflammatory cells were the fundamental source of inflammatory mediators in vivo. In our study, the classical ALI pathological changes were found in LPS-induced ALI murine model. However, the pathological changes were dose-dependently reduced by andrographolide. (Fig. 1, 3) Pulmonary edema, resulting in reduction of lung compliance and deterioration of pulmonary gas exchange, was other typical pathological changes in ALI. Endothelial injury associated microvascular leakage and reduction of pulmonary surfactant were considered as the mainly contributors of pulmonary edema. Our study found that andrographolide dose-dependently attenuated LPS-induced pulmonary edema in vivo. (Fig. 2) These observations indicated that andrographolide was capable of inhibiting the LPS-induced pathological changes in lung.

Neutrophils arrive quickly at sites of infection and form the first line of defence against invading micro-organisms. Neutrophil and macrophages were the major inflammatory cell types in ALI [27]–[29]. In LPS-induced inflammation, neutrophils and macrophages were activated by multiple inflammatory mediators, including GM-CSF, G-CSF, IL-8 and TNF-α. After activation, neutrophils and macrophages were recruited to the inflammation site by chemotactic factors, then, attached to the inflamed tissues, particularly endothelial cells, via adhesions, including L-selectin on neutrophils and E-selectin and P-selectin on endothelial cells [30]–[32]. Neutrophils and macrophages in BALF were significantly increased in LPS-induced ALI animal model. The significant recruitment of neutrophils and macrophages in murine ALI was diminished by andrographolide in the current study. MPO activity in pulmonary parenchyma, reflecting the activation of neutrophils, was largely up-regulated in ALI condition. Accordingly, MPO activity was also notably suppressed by andrographolide. (Fig. 6) These findings might partially explain that the reduction in neutrophils and macrophages and down–regulation of MPO activation could prevent the animals from developing ALI and implied that the occurrence of ALI was due to the continuous and cumulative damages from the activation of neutrophils and macrophages.

Various inflammatory mediators were involved in ALI. Among them, TNF-α, IL-6 and IL-1β were considered as the most important inflammatory mediators in innate immune response [33]–[36]. After LPS injection, LPS was quickly combined with an acute response protein (ARP), LPS binding protein (LBP). Then, the compound was identified by CD14, a membrane protein mainly expressed on inflammatory cells such as macrophages and neutrophils. Subsequently, LPS-LBP-CD14 compound activates inflammatory cells though TLRs family associated pathways, particularly TLR4, resulting in various oxidation enzymes synthesis, including MPO and elastase, leading to pathogen killing and tissue damage, along with more inflammatory mediators releasing which is called inflammatory cascade [30], [32]. According to our data, TNF-α, IL-6 and IL-1β in BALF were noticeably raised by LPS stimulation. Meanwhile, figure 5 also showed that andrographolide dose-dependently reduced the up-regulated TNF-α, IL-6 and IL-1β in BALF. These findings indicate that andrographolide effectively inhibited LPS-induced endothelial and epithelial injury and inflammatory cells infiltration in vivo, blocking the uncontrolled inflammatory process in ALI. Various inflammatory mediators were involved in ALI.

It is known that amount of inflammatory mediators and adhersion molecules (AM) participate in inflammation. VCAM-1 belongs to the immunoglobulin gene superfamily. Its main function is to mediate epithelial cells, including alveolar epithelial cells, binding to the leukocytes with VLA-4. Otherwise, Qiangsong Tong, et al found that on the upstream of the transcription start site of the VACM-1 gene contains a binding site for NF-κB [20]. Meanwhile, in another study, Qiangsong Tong, et al demonstrated that NF-κB was an important regulator on VEGF expression, which is considered to be essential in regulation vascular permeability in ALI associated pulmonary edema [21], [22]. Therefore, we assumed that VCAM-1 and VEGF can be used to detect activation of NF-κB signaling pathway in LPS-induced ALI. Otherwise, to confirm the anti-inflammatory properties of andrographolide, MEL-12 cells, a cell line derived from murine alveolar epithelial cells, were chosen in our investigation. As expected, MTT assay revealed that the reduced cell viability resulting from LPS stimulation was greatly improved by andrographolide. Meanwhile, there is convincing evidence that VCAM-1 and VEGF were up-regulated by LPS stimulation in vivo and in vitro. Furthermore, our findings also found that the overexpressed VCAM-1 and VEGF were inhibited by andrographolide, both in vivo and in vitro.

Then, it is reasonable to explore the condition of NF-κB family members and their relations with andrographolide in our study. The NF-κB family of transcriptional factors (TF) plays a critical role on regulation the expression of a wide variety of genes, particularly in the inflammatory process. The NF-κB family has five cellular members. They are p105/p50 (NF-κB1), p100/p52 (NF-κB2), p65 (RelA), RelB and c-Rel [1], [17]–[19]. The activation of NF-κB pathway was tightly dependent on the IKK (IκB kinase) complex, which consists of the regulatory subunit, IKKγ also called NF-κB essential modifier (NEMO), and the catalytic subunits, IKKα and IKKβ. And its downstream substrate is IκBα. In unstimulated cells, NF-κB p50/NF-κB p65 dimer is bound to IκBα. After the stimulation, IKKα and IKKβ are phosphorylated, then, promoting the phosphorylation of IκBα. Rapidly, phosphorylated IκBα is degraded via the ubiquitin-proteasome pathway. The degradation of IκBα leads to NF-κB p50/NF-κB p65 dimer phosphorylation and translocation, resulting in the transcription of genes, finally [1], [17]–[19]. In animal model, our results showed that LPS injection markedly promoted NF-κB p65 phosphorylation and significantly enhanced NF-κB p65 DNA binding activity. However, LPS-induced over phosphorylated NF-κB p65 and enhanced NF-κB p65 DNA binding activity both were notably suppressed by andrographolide in vivo. Then, to gain insight into the biochemical mechanism, we studied the effects of andrographolide on LPS-induced activation of NF-κB pathway in MEL-12 cells. The molecules, including IKKβ, IΚBα and NF-κB p65, in NF-κB pathway were analyzed in vitro. Our data showed that LPS led to rapidly activation of NF-κB pathway, involving IKKβ phosphorylation, IκBα phosphorylation and NF-κB p65 phosphorylation. Nevertheless, the LPS-induced phosphorylated proteins were all attenuated by andrographolide administration. Furthermore, andrographolide also compromised NF-κB p65 DNA binding activity in vitro. These results demonstrated that the anti-inflammatory property of andrographolide is very likely mediated by inactivation of NF-κB at the level of the phosphorylation of IKKβ.

### Conclusion

Our data show, to our knowledge for the first time, that andrographolide treatment reduced the severity of LPS-induced ALI, more likely by virtue of andrographolide-mediated NF-κB inhibition at the level of IKKβ activation. These results suggest andrographolide may be considered as an effective and safe drug for the potential treatment of ALI. Nevertheless, animal studies are limited to hours while ARDS patients often are treated for several days or even weeks. Taken together, we believe that andrograophlide may show its beneficial effects over time when it is given at the beginning of ALI.
